# Identification and validation of chemokine system-related genes in idiopathic pulmonary fibrosis

**DOI:** 10.3389/fimmu.2023.1159856

**Published:** 2023-04-14

**Authors:** Tianming Zhao, Xu Wu, Xuelei Zhao, Kecheng Yao, Xiaojuan Li, Jixiang Ni

**Affiliations:** ^1^ Department of Respiratory and Critical Care Medicine, The People’s Hospital of China Three Gorges University, The First People’s Hospital of Yichang, Yichang, China; ^2^ Department of Gastroenterology, The People’s Hospital of China Three Gorges University, The First People’s Hospital of Yichang, Yichang, China; ^3^ Department of Geriatrics, The People’s Hospital of China Three Gorges University, The First People’s Hospital of Yichang, Yichang, China

**Keywords:** chemokine, idiopathic pulmonary fibrosis, gene signature, immune infiltration, biomarker

## Abstract

**Background:**

Idiopathic pulmonary fibrosis (IPF) is a chronic progressive interstitial lung disease with limited therapeutic options. Recent studies have demonstrated that chemokines play a vital role in IPF pathogenesis. In the present study, we explored whether the gene signature associated with chemokines could be used as a reliable biological marker for patients with IPF.

**Methods:**

Chemokine-related differentially expressed genes (CR-DEGs) in IPF and control lung tissue samples were identified using data from the Gene Expression Omnibus database. A chemokine-related signature of the diagnostic model was established using the LASSO-Cox regression. In addition, unsupervised cluster analysis was conducted using consensus-clustering algorithms. The CIBERSORT algorithm was used to calculate immune cell infiltration across patient subgroups. Finally, we established a mouse model of bleomycin-induced pulmonary fibrosis and a model of fibroblasts treated with TGFβ1. Expression levels of chemokine-related signature genes were determined using real-time quantitative polymerase chain reaction (RT-qPCR).

**Results:**

We established a chemokine-related eleven-gene signature of a diagnostic model consisting of CXCL2, CCRL2, ARRB1, XCL1, GRK5, PPBP, CCL19, CCL13, CCL11, CXCL6, and CXCL13, which could easily distinguish between IPF patients and controls. Additionally, we identified two subtypes of IPF samples based on chemokine-related gene expression. Pulmonary function parameters and stromal scores were significantly higher in subtype 1 than in subtype 2. Several immune cell types, especially plasma cells and macrophages, differ significantly between the two subtypes. RT-qPCR results showed that the expression levels of *Cxcl2* and *Ccl2* increased considerably in bleomycin-induced mice. Meanwhile, *Arrb1*, *Ccrl2*, *Grk5*, and *Ppbp* expression was significantly reduced. Furthermore, multiple chemokine-related genes were altered in TGFβ1 or TNFα-induced fibroblast cells.

**Conclusions:**

A novel chemokine-related eleven-signature of diagnostic model was developed. These genes are potential biomarkers of IPF and may play essential roles in its pathogenesis.

## 1 Introduction

Idiopathic pulmonary fibrosis (IPF) is a chronic progressive lung disease characterized by pulmonary scarring (1). The incidence of IPF is increasing, and IPF is a leading cause of death in an aging population (2). The prognosis for IPF is often poor, with an average survival of 3–5 years after diagnosis (1). The incidence and prevalence of IPF were analyzed as 0.09–1.30 per 10,000 and 0.33–4.51 per 10,000, respectively (3). The treatment options for IPF are limited. Pirfenidone and nintedanib are currently FDA-approved oral agents that reduce IPF progression (4, 5). Thus, identifying specific biomarkers, especially for early stage and prompt therapy, is vital for improving the prognosis of patients with IPF.

The pathogenesis of IPF involves multiple environmental risk factors and multi-gene alterations that contribute to epithelial cell damage and apoptosis, recruit immune cells to the site of injury, and activate fibroblasts to secrete extracellular matrix to initiate repair (6). Chemokines play an essential role in injury and repair (7). Chemokines are small-molecular-weight proteins secreted by multiple cell types and are involved in the process of pulmonary fibrosis (8–10). For example, alveolar epithelial cell (AECs) injury increases the expression of both CCL2 and CCL12, and alveolar epithelial cell-specific deletion of CCL12 prevents pulmonary fibrosis in mice, but not in CCL12 null mice. Loss of CCL12 in AECs leads to decreased macrophage recruitment (10). During pulmonary fibrosis, alveolar macrophages secrete multiple chemokines such as CCL18 and CCL1, which directly activate pulmonary fibroblasts and stimulate collagen production (8, 11). CCL18 levels significantly increase in the serum and bronchoalveolar lavage fluid (BALF) of patients with multiple interstitial lung diseases, including IPF (12). Serum CCL18 concentrations >150 ng/mL significantly increased mortality in patients with IPF, indicating that serum CCL18 concentrations are a good predictor of IPF (13). Neutralizing CCL1 or inhibiting CCL1 signaling reduces pulmonary fibrosis *in vitro* and *in vivo*, indicating that CCL1 is a potential therapeutic target for IPF (8). In IPF, various cells express chemokines and chemokine receptors, constituting a complex chemokine system that regulates the pathogenesis of IPF. Thus, the role of chemokine system-related genes in the diagnosis and prognosis of IPF remains unclear and requires further investigation.

In the present study, we identified chemokine-related differentially expressed genes (CR-DEGs) in the control and IPF samples. Based on the least absolute shrinkage and selection operator (LASSO), eleven CR-DEGs were included to establish a diagnostic model in the training and validation sets. In addition, IPF samples were divided into two subgroups based on eleven genes and characterized for each subgroup. Finally, we examined the expression of eleven CR-DEGs in bleomycin-induced injury and TGFβ1-induced pulmonary fibroblasts.

## 2 Materials and methods

### 2.1 Data source and processing

The gene expression matrix and clinical data of IPF samples were downloaded from the NCBI GEO database (http://www.ncbi.nlm.nih.gov/geo/) (14). The training cohort consisted of 122 IPF and 91 control samples from GSE47460 [GPL14550 platform]. The validation cohort comprised 112 IPF and 20 control samples from GSE70866 [GPL14550 platform]. GSE47460 [GPL6480 platform] served as a validation cohort. One hundred thirteen chemokine-related genes (CRGs) were extracted from GeneCards (https://www.genecards.org/).

### 2.2 Identification of the CR-DEGs

Differentially expressed genes (DEGs) were extracted between the IPF and normal samples using the “limma” R package (the absolute value of log2 fold change (log2FC) was more significant than 1, and false discovery rate (FDR) was less than 0.05). We compared the differences between DEGs and CRGs and retained the pooled portion as CR-DEGs. The “pheatmap” R package and “cor” functions were used to draw heatmaps and calculate the correlation between the CR-DEGs. Protein-protein interactions (PPI) between CR-DEGs were predicted using the STRING database. Cytoscape Version 3.0.0 was used to visualize the PPI network (15). Next, we enriched GO biological processes and KEGG signaling for genes in the network using Cluster Profiler version 4.4.4 (16).

### 2.3 Construction of diagnostic models

Based on the expression levels of CR-DEGs in the GSE47460 [GPL14550 platform] cohort, “rms” R packages were used for univariable logistic regression. We then used LASSO to screen for optimal CR-DEGs. For the GSE47460 [GPL14550 platform] cohort, a CR-DEG-based diagnostic classifier was constructed using the Support Vector Machine (SVM) method. The diagnostic model was evaluated using the ROC curve method in the training cohort (GSE47460 [GPL14550 platform]) and two independent validation cohorts (GSE70866 and GSE47460 [GPL6480 platform]).

### 2.4 Clinical relevance of CR-DEGs

Clinical information, including age, diffusion capacity of carbon monoxide (dlco), forced vital capacity (fvc), and forced expiratory volume in 1 s (fev1), was extracted from the GSE47460 dataset. Fisher’s exact test was used for categorical variables to analyze the distribution of clinical information in the samples. The Kruskal–Wallis test was used to compare continuous data variables, and correlations between the expression levels of CR-DEGs and clinical parameters were calculated using the cor function.

### 2.5 Analysis of immune infiltration

We used CIBERSORT to estimate the proportional immune cell types in the GSE47460 [GPL14550 platform] samples. We then compared the variability of immune cell distribution in the IPF and control groups using the Kruskal–Wallis test. Correlations between the expression levels of CR-DEGs, which were used to construct diagnosis models and immune cells, were calculated using a cor function.

### 2.6 Prognostic relevance of diagnostic CR-DEGs

We obtained survival information from the GSE70866 cohort to observe the prognostic relevance of diagnostic CR-DEGs. The IPF samples were divided into two groups (high- and low-expression groups) according to the median gene expression value. The prognostic difference between the high- and low-expression groups was assessed using the Kaplan–Meier curve method (survival package) in the R language, and the p-value was calculated using the log-rank test.

### 2.7 Analysis of molecular subtype

Based on the expression levels of CR-DEGs constructed for diagnostic models, we performed a consensus clustering analysis of the IPF samples from the GSE47460 [GPL14550 platform] cohort using the “Consensus ClusterPlus” package. The “GSVA” package assessed the chemokine scores for each IPF sample, and then the chemokine scores of the different subtypes were compared using the Kruskal–Wallis test. As described above, we also compared the differences in clinical parameters, immune infiltration, and stromal scores between the subtypes.

### 2.8 Pathway enrichment analysis in subgroups

All KEGG data were downloaded from the GSEA database. We then quantified each KEGG pathway using the “GSVA” package based on the gene expression levels in the GSE47460 [GPL14550 platform] sample. We then used the “limma” package to screen for differentially expressed genes between subgroups (FDR<0.05 and |log2FC|>0.263). The ClusterProfiler” package was used to perform Gene Ontology (GO), including biological processes (BP), and Kyoto Encyclopedia of Genes and Genomes (KEGG) pathway enrichment analysis.

### 2.9 Animal model

Eight-week-old male C57BL/6J mice were purchased from the SLRC Laboratory Animal Company (Hunan, China). All animals were kept in an SPF environment at China Three Gorges University. The animal experimental protocol was approved by the Ethics Committee of China Three Gorges University (Approval No. 2022B100A). The animal model of lung fibrosis was established by a single intratracheal administration of bleomycin (2 U/kg, Hisun Pharmaceutical, China) or an equal amount of saline as a control. Mice were sacrificed 21 days after establishment of the mouse model. The body and lung weights were measured. The left lung was embedded in paraffin and stained with Masson’s trichrome. The right lung was collected and frozen in liquid nitrogen for real-time quantitative polymerase chain reaction (RT-qPCR).

### 2.10 Cells culture and treatment

The human embryonic lung fibroblast MRC-5 cell line was purchased from Procell (Wuhan, China) and cultured in Minimum Essential Medium (MEM, Procell) containing non-essential amino acids, 10% fetal bovine serum (FBS, VivaCell) and 1% penicillin-streptomycin (VivaCell) at 37°C with 5% CO_2_. MRC-5 cells were treated with TGFβ1 (5 ng/mL), and TNFα (10 ng/mL) for 24 h.

### 2.11 RT-qPCR

The expression of these core CR-DEGs in fibroblasts stimulated with TGF-β1 and TNF-α or bleomycin-treated lung tissue was further verified using RT-qPCR. Total RNA was isolated using TRIZOL. The cDNA was synthesized using a cDNA synthesis kit (Vazyme, Nanjing, China). RT-qPCR was performed using the Taq Pro Universal SYBR qPCR Master Mix (Vazyme, Nanjing, China). *GAPDH* or *ACTB* was used as the reference gene. The relative fold-change was calculated using the 2^−△△Ct^ method.

### 2.12 Western blot analysis

Western blot analysis was performed to detect protein level of GRK5, ARRB1, and CCRL2 in fibroblasts stimulated with TGF-β1 and TNF-α. Briefly, MRC-5 cells were lysed on ice using radioimmune precipitation assay (RIPA) lysis buffer (Servicebio, Wuhan, China). Protein concentration was measured using BCA protein assay kit. Protein extracts (20 ug) were separated by SDS-polyacrylamide gels (SDS-PAGE), transferred to PVDF membranes. Membranes were then incubated with primary antibodies against α-SMA (1:1000, Cell Signaling Technology, MA, USA), FN1 (1:2000, Proteintech, Wuhan, China), Tubulin (1:2000, Santa Cruz, CA, USA), GRK5 (1:1000, Proteintech, Wuhan, China), ARRB1 (1:1000, Proteintech, Wuhan, China), CCRL2 (1:3000, Proteintech, Wuhan, China) overnight at 4°C. Horseradish peroxidase (HRP)-conjugated anti-mouse or anti-rabbit whole IgG secondary antibodies (1:5000, Birmingham, AL, USA) was identify the primary antibody. Protein bands were detected using Super ECL Star kit (US Everbright, Suzhou, China).

### 2.13 Statistical analysis

Statistical analysis was performed using the R software (version 3.6.1) and GraphPad Prism software (version 8.0). Different R packages were used to analyze the gene expression profiles. Data from RT-qPCR were presented as “mean ± standard error of mean (SEM)” or “mean ± standard deviation (SD)”. An Independent samples t-test was used to compare the treatment and control groups. Statistical significance was set at p<0.05.

## 3 Results

### 3.1 Identification of chemokine-related differentially expressed genes

**Figure 1:**
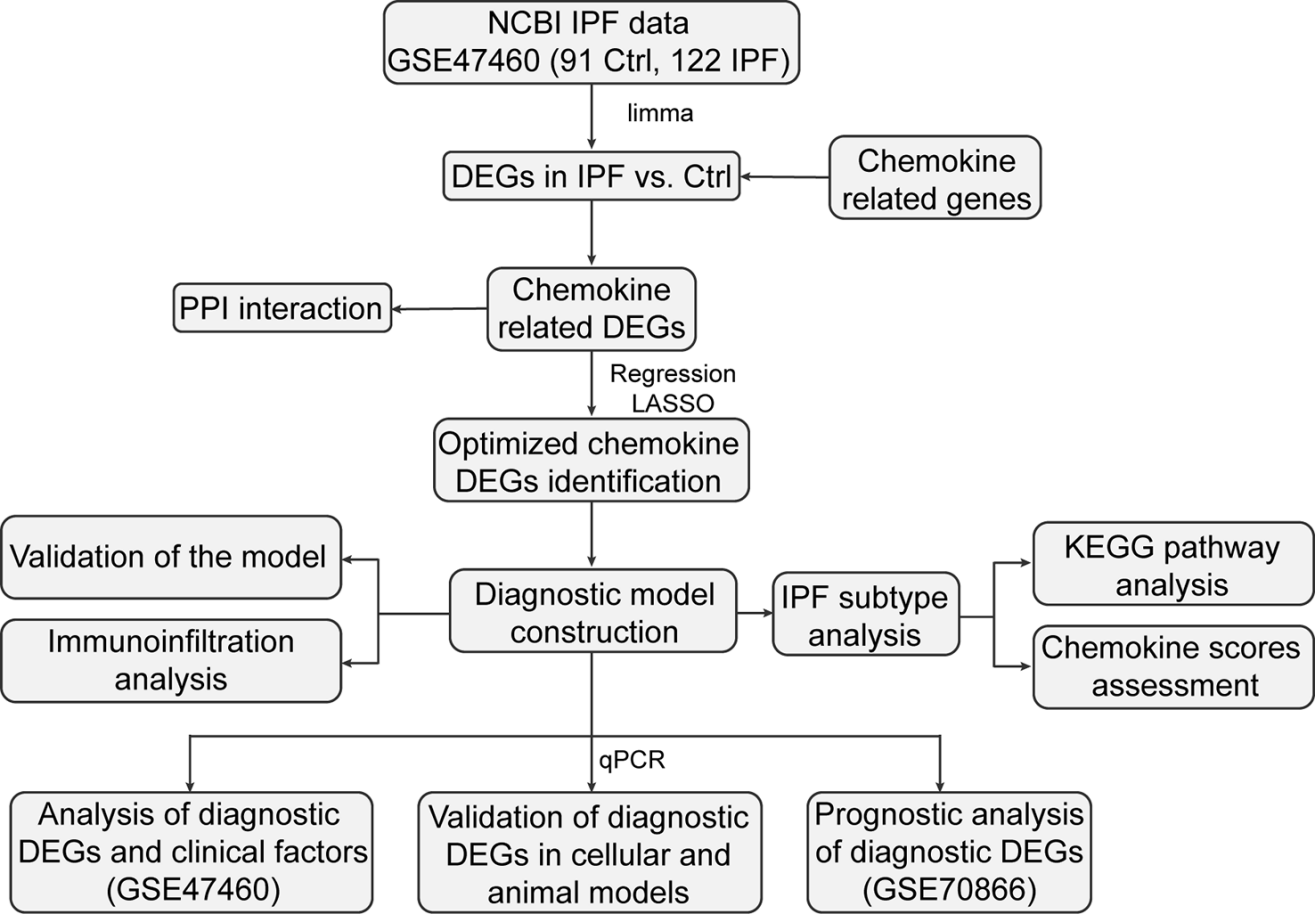
Flow diagram of this study.

**Figure 2:**
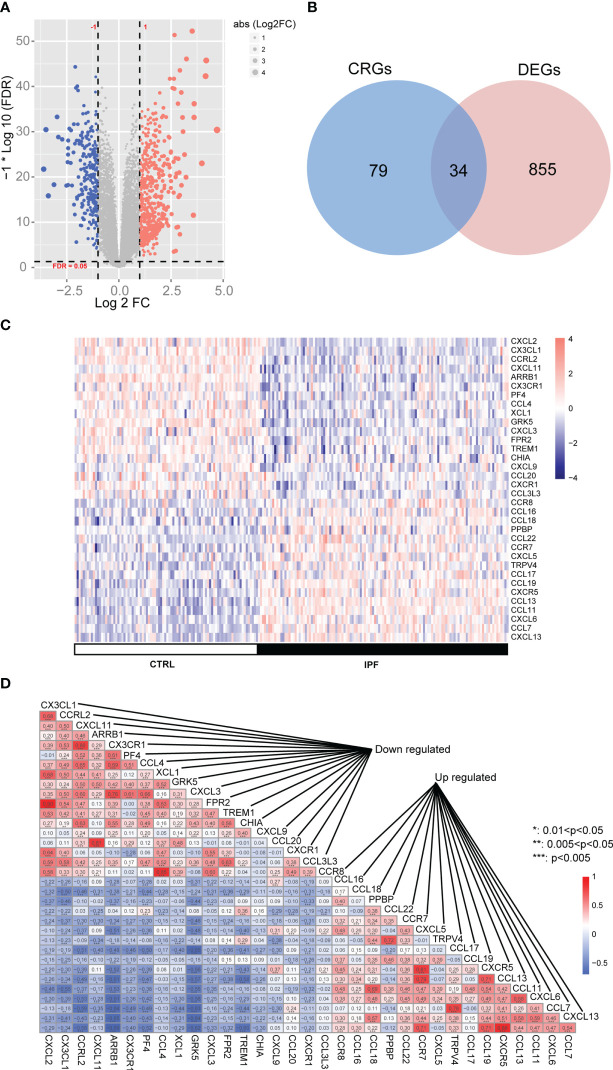
The differentially expressed chemokine-related genes (CRs) in IPF cohort. **(A)** Volcano plot of differentially expressed genes (DEGs). **(B)** Venn diagram of DEGs and CRs. **(C)** Heatmap of CR-DEGs in IPF samples. **(D)** Heatmap of correlation between CR-DEGs.

The workflow is illustrated in **Figure 1**. We investigated the DEGs in 122 IPF and 91 normal tissue samples (CTRL) from GS47460 [GPL14550 platform]. These specimens were obtained surgically and were diagnosed as having IPF or being controls by clinical history, CT scan, or surgical pathology. The baseline characteristics of the IPF and control individuals are shown in **Table 1**. As shown in the volcano map, 891 DEGs (306 downregulated and 585 upregulated) were identified (**Figure 2A**). In total, 113 CRGs were retrieved from the GeneCards database (**Table S1**). Thirty-four CR-DEGs were identified by investigating the intersection of the DEGs and CRGs (**Figure 2B**). The heatmap showed 34 CR-DEGs in IPF and control samples (**Figure 2C**). Chemokine receptors often play an essential role in the binding of specific chemokines. Based on this, we performed a correlation analysis between the CR-DEGs (**Figure 2D**). Additionally, we constructed a PPI network of CR-DEGs using the STING database. Among these CR-DEGs, CCL13, CCL11, CCL7, CXCL13, GRK5, ARRB1, CCRL2, and CXCL2 with the highest degrees were considered hub genes in this network (**Figure S1**). Biological processes of GO enrichment analysis revealed that CR-DEGs were mainly involved in cell chemotaxis, response to chemokines, chemokine-mediated signaling pathways, etc. (**Figure S2A**). The enrichment analysis of KEGG pathways included the chemokine signaling pathway, cytokine-cytokine receptor interaction, and IL-17 signaling pathway (**Figure S2B**).

**Table 1:** The baseline characteristics of the study subjects.

Clinical features	IPF (N=122)	CTRL (N=91)	*P value*
Gender			0.0013
Female	41	51	
Male	81	40	
Age	64.51 ± 8.40	63.79±11.49	0.615
Predicted dlco (%)	49.51±18.73	82.86±16.44	< 0.001
Predicted fev1 (%)	71.05±17.51	94.58±12.97	< 0.001
Predicted fvc (%)	64.28±14.62	94.46±13.11	< 0.001

### 3.2 Construction of diagnostic model based on chemokine-related hub genes

**Figure 3:**
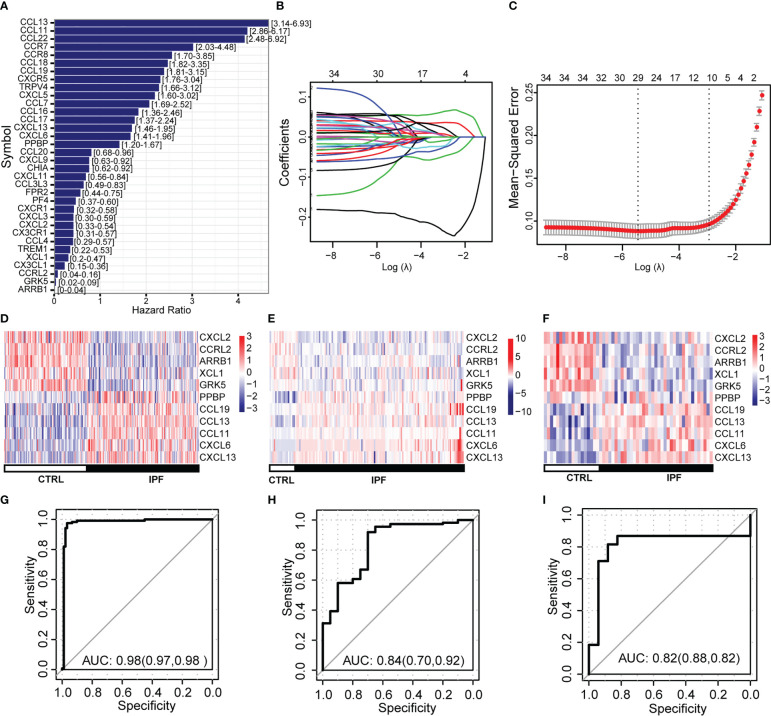
Construction and validation of diagnostic models based on CR-DEGs. **(A)** The logistic regression analysis of CR-DEGs. **(B)** Diagnostic model construction using a least absolute shrinkage and selection operator (LASSO) Cox regression model. **(C)** Coefficient distribution plots to select the optimum lambda value. **(D–F)** Heatmap of the gene-expression profiles of model-related CR-DEGs in the training **(D)** and external validation cohorts **(E, F)**. **(G–I)** ROC curves of diagnostic models in training **(G)** and external validation cohorts **(H, I)**.

**Figure 4:**
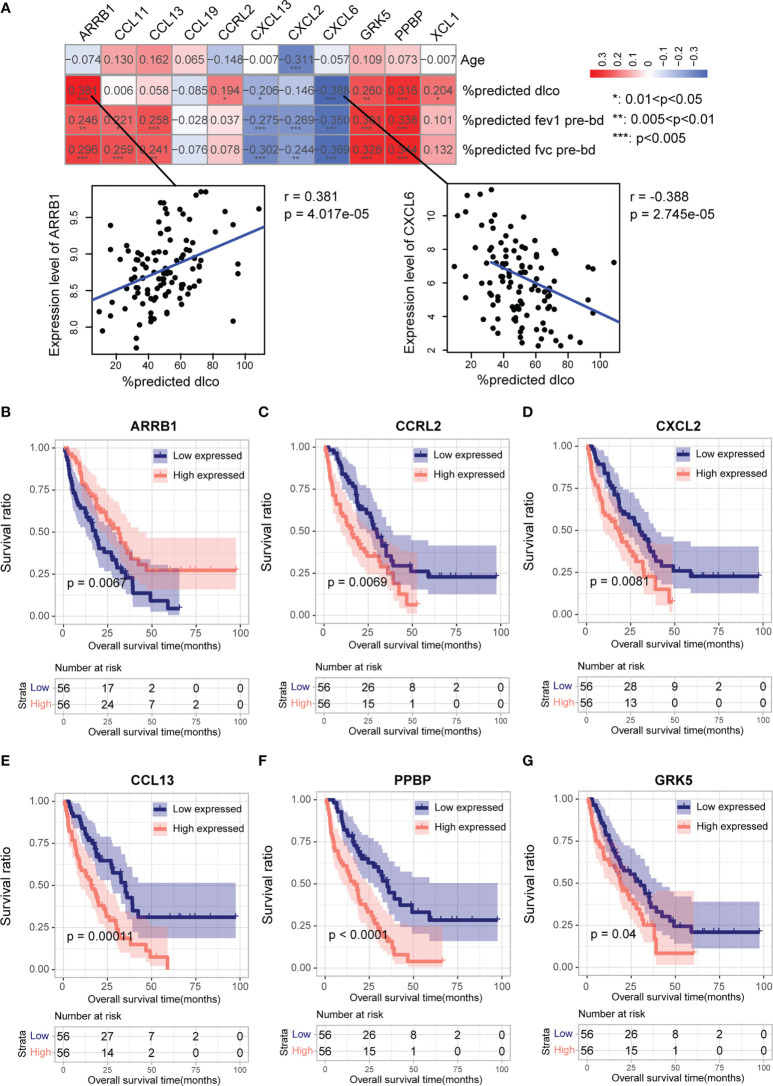
Association of clinical parameters and prognostic value of eleven CR-DEGs in IPF patients. **(A)** Correlation between eleven CR-DEGs and age, dlco, fev1, and fvc. **(B–G)**. K-M analysis and the predictive value of ARRB1 **(B)**, CCRL2 **(C)**, CXCL2 **(D)**, CCL13 **(E)**, PPBP **(F)**, and GRK5 **(G)** for the survival in the BALF of patients with IPF.

We performed univariate logistic regression analysis of 34 CR-DEGs (**Figure 3A**). Next, we used LASSO to screen for optimal CR-DEGs. Eleven CR-DEGs were subjected to LASSO Cox regression analysis to construct a diagnostic model (**Figures 3B, C**). The heatmap showed that eleven CR-DEGs in the diagnostic model had significant differences in precision between the IPF and control samples in the training cohort (GS47460 [GPL14550 platform]) (**Figure 3D**) and two external validation cohorts (GSE70866 and GS47460 [GPL6480 platform]) (**Figures 3E, F**). **Figures 3G–I** show the ROC curves of the eleven CR-DEGs diagnostic models in the GS47460 [GPL14550 platform], GSE70866, and GS47460 [GPL6480 platform] datasets, respectively. Eleven CR-DEGs were significantly associated with clinical parameters in patients, such as age, dlco, fev1, and fvc (**Figure 4A**). For example, ARRB1 expression was significantly positively correlated with dlco, indicating that higher ARRB1 expression is associated with better lung function. In contrast, CXCL6 expression was significantly negatively correlated with dlco, suggesting that CXCL6 is a marker of poor lung function (**Figure 4A**). Therefore, these results suggest that the eleven CR-DEGs signatures may serve as potential diagnostic factors in IPF patients. Based on the median values, patients with IPF from the GSE70866 cohort were divided into two groups. The association between gene expression and outcome was assessed using Kaplan–Meier curves. Six genes (ARRB1, CCRL2, CXCL2, CCL13, PPBP, and GRK5) showed a prognostic value. In bronchoalveolar lavage (BAL) cells, high expression of CCRL2, CXCL2, CCL13, PPBP, and GRK5 was significantly associated with shorter survival times. High ARRB1 expression was associated with better outcomes (**Figures 4B–G**).

### 3.3 CR-DEGs and immune cell infiltration

**Figure 5:**
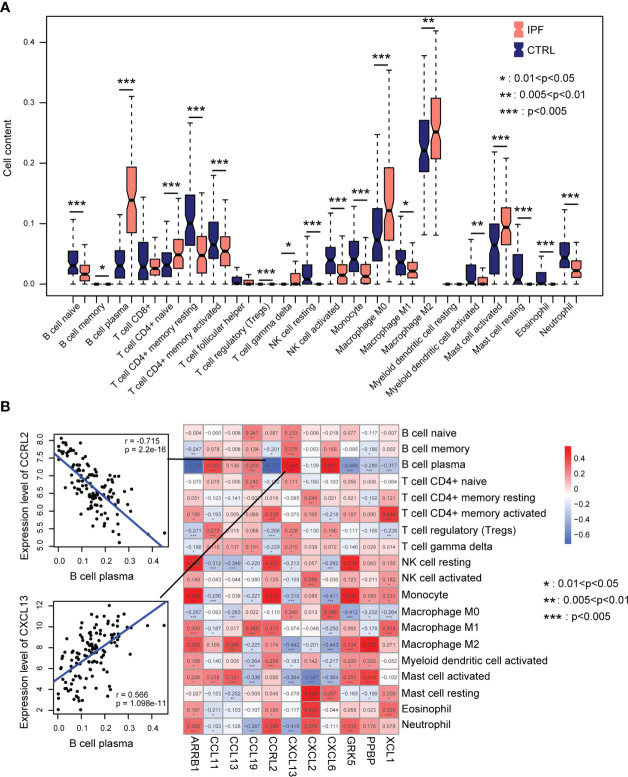
Immune cell infiltration. **(A)** Distribution of immune cells in normal and IPF lung tissue. **(B)** Correlation between eleven CR-DEGs expression levels and levels of infiltration immune cells.

The CIBERSORT algorithm was used to assess the immune microenvironment of the GSE GS47460 [GPL14550 platform] dataset. A boxplot indicated that the infiltration of multiple immune cells was significantly different between IPF and control samples (**Figure 5A**). For example, plasma cells, naïve CD4^+^ T cells, macrophages (M0 and M2), and activated mast cells infiltrated significantly higher levels in the IPF tissue (**Figure 5A**). Next, we explored the relationship between eleven CR-DEGs and immune cell infiltration. The results suggest that eleven CR-DEGs were significantly associated with the infiltration level of immune cells (**Figure 5B**).

### 3.4 Subtype based on the diagnosis of CR-DEGs

**Figure 6:**
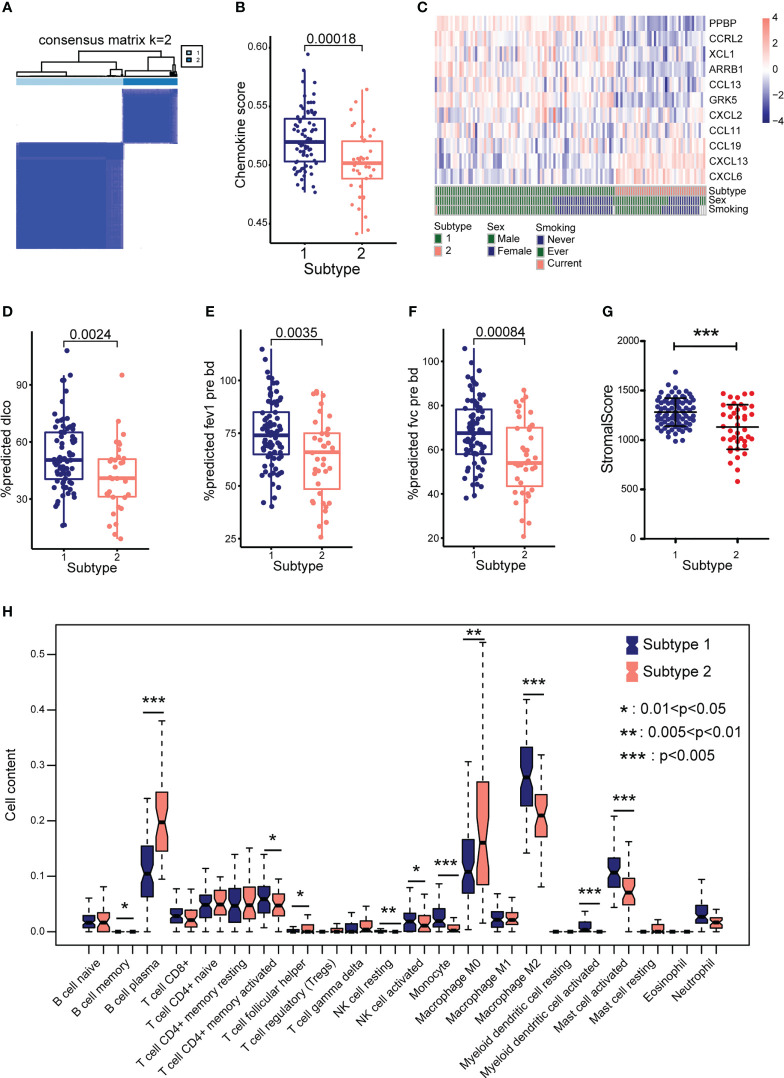
Identify different subtypes based on eleven CR-DEGs. **(A)** Hierarchical map of subtype analysis. **(B)** The chemokine score between the two subtypes. **(C)** Distribution of the eleven CR-DEGs in GSE47460 cohorts in subtype, sex, and smoking. **(D)** The pulmonary function parameters, dlco **(D)**, fev1 **(E)**, and fvc **(F)** in two subtypes. **(G)** Stromal score in two subtypes. **(H)** The proportion of 22 types of immune cells in two subtypes.

To explore the biological characteristics of chemokine-related genes, IPF samples were classified into two clusters (subtype 1 and subtype 2) using the “ConsensusClusterPlus” package in R, according to eleven CR-DEGs expression levels. Subtypes 1 and 2 contained 81 and 41 IPF samples, respectively (**Figure 6A**). The chemokine score for each sample was assessed using the GSVA algorithm. As shown in **Figure 6B**, the chemokine score was significantly higher for subtype 1 than for subtype 2. The heatmap shows the expression patterns of eleven CR-DEGs in subtypes 1 and 2 (**Figure 6C**). Pulmonary function parameters (dlco, fev1, and fvc) and stromal scores were significantly higher in subtype 1 than in subtype 2 (**Figures 6D–G**). In addition, immune infiltration analysis showed that the levels of infiltration of multiple immune cells differed significantly between the two subtypes (**Figure 6H**).

### 3.5 Functional enrichment analysis

**Figure 7:**
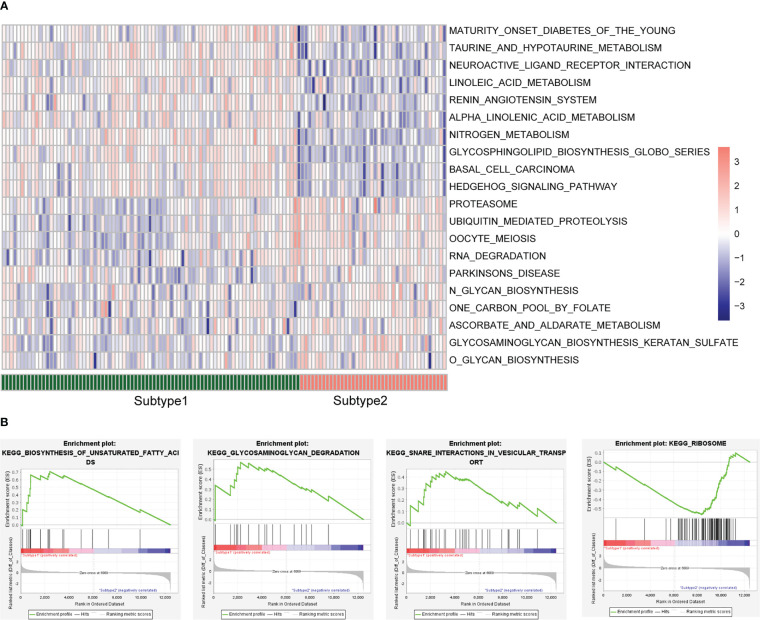
Enrichment analysis between the two subtypes. **(A)** GSVA enrichment analysis. **(B)** GSEA enrichment analysis.

To further investigate changes in biological function between subtypes based on the expression levels of the eleven CR-DEGs, we performed GSEA for subtypes 1 and 2. The significantly enriched pathways in subtypes 1 and 2 are shown in **Figure 7A**. Subsequently, GSEA was performed for these two subtypes. The results indicated that the samples in subtype 1 were significantly enriched in unsaturated fatty acid biosynthesis, glycosaminoglycan degradation, and snare interactions in vesicular transport. Subtype 2 was significantly enriched in ribosomes (**Figure 7B**).

### 3.6 Validation of model gene expression in TGFβ1- or TNFα-treated lung fibroblast cells

**Figure 8:**
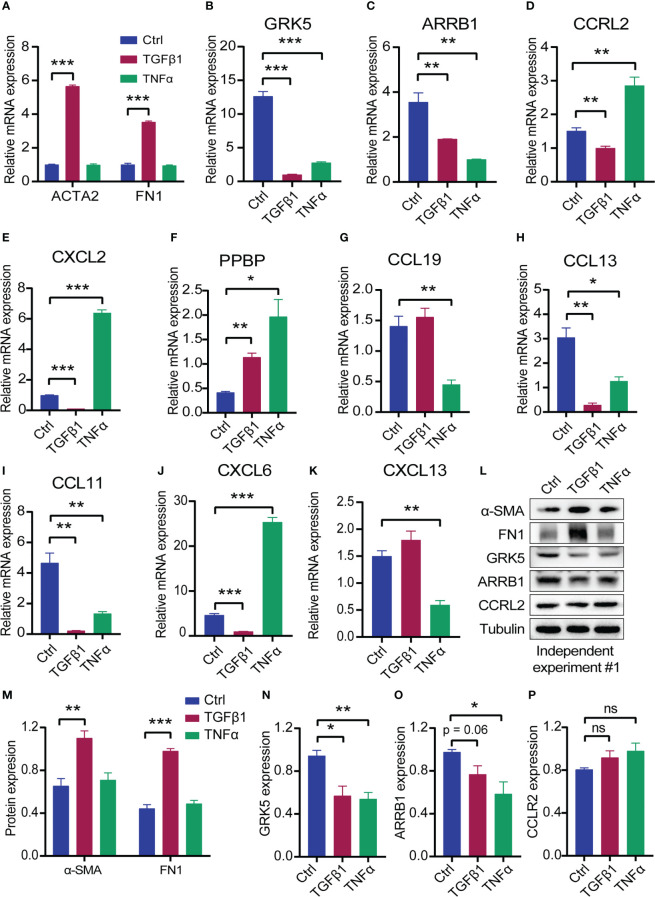
The expression of the eleven CR-DEGs in MRC-5 treated with TGFβ1 or TNFα for 24 h. **(A)** mRNA levels of ACTA2 and FN1 in control, TGFβ1, and TNFα group. **(B–K)** mRNA levels of CXCL2 **(B)**, CCRL2 **(C)**, ARRB1 **(D)**, GRK5 **(E)**, PPBP **(F)**, CCL19 **(G)**, CCL13 **(H)**, CCL11 **(I)**, CXCL6 **(J)**, and CXCL13 **(K)** in control, TGFβ1, and TNFα group, **(L)** Protein levels of α-SMA, FN1, GRK5, ARRB1, and CCRL2. **(M-P)** Quantitative analysis of α-SMA, FN1 **(M)**, GRK5 **(N)**, ARRB1 **(O)**, and CCRL2 **(P)**. Values were expressed as mean ± SEM, *p < 0.05, **p < 0.01, ***p < 0.001. t test was used. Ctrl, control; ns, not significant difference.

Upon stimulation with various cytokines, pulmonary fibroblasts are transformed into myofibroblasts that synthesize large amounts of the extracellular matrix. TGF-β1 and TNF-α, well-known profibrotic and proinflammatory cytokines, are widely used to induce fibroblast-to-myofibroblast transformation (17, 18). The mRNA levels of the eleven CR-DEGs were estimated using RT-qPCR. Primer sequences are listed in **Table S2**. Treatment of MRC-5 cells with TGF-β1 (5 ng/mL for 24 h) significantly upregulated ACTA2 (encoding α-SMA) and FN1 (encoding fibronectin) expression (**Figure 8A**), suggesting that TGF-β1 promotes fibroblast-to-myofibroblast transformation. Additionally, the expression of several chemokines and receptors decreased, including CXCL2, CCRL2, ARRB1, GRK5, CXCL6, CCL13 (human only), and CCL11. Stimulation of MRC-5 cells with TNF-α (10 ng/mL for 24 h) resulted in a significant increase in CXCL2, CCRL2, and CXCL6 expression, whereas ARRB1, GRK5, CCL13, CCL11, CCL19, and CXCL13 expression was significantly decreased (**Figures 8B–K**). XCL1 was not detected in the MRC-5 cells. Western blot revealed elevated of α-SMA and FN1, as well as down-regulated GRK5, ARRB1 in TGFβ1-treated lung fibroblasts (**Figures 8L–P**, **Figure S3**).

### 3.7 Validation of model gene expression in bleomycin induced-pulmonary fibrosis model

**Figure 9:**
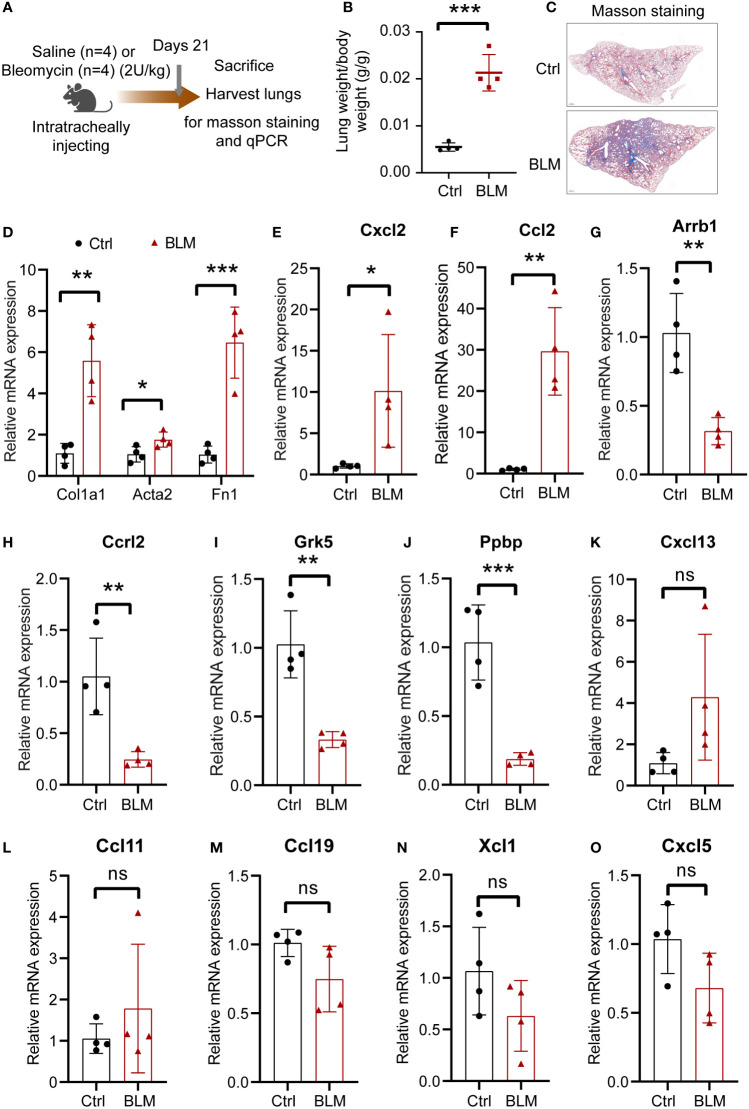
The expression of the eleven CR-DEGs in bleomycin-induced lung fibrosis. **(A)** Schematic diagram of bleomycin-induced lung fibrosis. **(B)** Lung index (lung weight/body weight). **(C)** Representative images of Masson’s trichrome staining of the lung section. **(D)** mRNA levels of Col1a1, Acta2, and Fn1 in lung tissue. **(E-O)** mRNA levels of Cxcl2 **(E)**, Ccl2 **(F)**, Arrb1 **(G)**, Ccrl2 **(H)**, Grk5 **(I)**, Ppbp **(J)**, Cxcl13 **(K)**, Ccl11 **(L)**, Ccl19 **(M)**, Xcl1 **(N)**, and Cxcl5 **(O)** in control and bleomycin group.. Values were expressed as mean ± SD (n = 4), *p < 0.05, **p < 0.01, ***p < 0.001. t test was used. Ctrl, control; BLM, bleomycin; ns, not significant difference.

Bleomycin-induced murine pulmonary fibrosis is the most representative and commonly used experimental model for IPF studies (19). We further validated model gene expression in BLM-induced pulmonary fibrosis. We established a model of pulmonary fibrosis by intratracheal injection of bleomycin (2 U/kg). The mice were sacrificed on day 21, and lung tissues were harvested (**Figure 9A**). The lung index was significantly increased in mice treated with bleomycin (**Figure 9B**). Masson staining showed that bleomycin significantly increased collagen deposition (**Figure 9C**). The fibrosis marker genes *Col1a1*, *Acta2*, and *Fn1* were also significantly upregulated in bleomycin-induced mice (**Figure 9D**). These results suggest that the pulmonary fibrosis model was successfully established. Next, validation of the model genes mRNA levels was validated using RT-qPCR. Primer sequences are listed in **Table S2**. The results demonstrated that Cxcl2 and Ccl2 (human homolog CCL13) expression were significantly increased in bleomycin-induced mice (**Figures 9E, F**). Meanwhile, Arrb1, Ccrl2, Grk5, and Ppbp levels were significantly reduced (**Figures 9G–J**). Cxcl13, Ccl11, Ccl19, Xcl1, and Cxcl5 (human homolog CXCL6) mRNA levels did not change between the two groups of mice (**Figures 9K–O**).

## 4 Discussion

Idiopathic pulmonary fibrosis is a chronic, progressive, and fatal disease characterized by alveolar epithelial injury, immune cell recruitment, and fibroblast activation despite the application of anti-fibrotic therapy. Accumulating evidence indicates that injured epithelial cells and abnormally activated macrophages secrete chemokines that induce fibroblast expansion and activation (6). Chemokine and chemokine receptor signals play a role in cell migration, activation, and response to lung injury repair, including IPF (8, 10, 20, 21). Antibodies that neutralize chemokines may contribute to treating IPF (8). In the lung tissues, many cells secrete chemokines or express chemokine receptors. The analysis of chemokines and receptors may be conducive to diagnosing and treating IPF, even when evaluating the prognosis of patients with IPF. Therefore, establishing a multi-gene diagnostic model for patients with IPF based on the chemokine system is necessary.

In the present study, 34 CR-DEGs were screened from the GSE47460 dataset, containing 112 IPF and 91 control samples. Eleven CR-DEGs signatures were identified using the LASSO regression model to construct a diagnosis IPF model. The diagnosis model easily distinguished between IPF and control samples, highlighting that the chemokine system differs between patients with IPF and control individuals. In addition, we explored the prognostic role of the eleven IPF genes in the GSE70866 dataset. The results suggest that ARRB1, CCRL2, CXCL2, CCL13, PPBP, and GRK5 are better factors for determining the prognosis of IPF. Furthermore, consensus clustering was used to classify patients with IPF into two subgroups based on the expression levels of eleven CR-DEGs. Finally, we evaluated the expression of eleven CR-DEGs in a bleomycin-induced pulmonary fibrosis model and in TGFβ1-activated human lung fibroblasts. Although preclinical models of bleomycin-induced pulmonary fibrosis are the most commonly experimental models to investigate the mechanisms of pulmonary fibrosis, there are differences with IPF, such as recovery from bleomycin-induced pulmonary fibrosis. Consistent with the transcriptome data of IPF lung tissue, Arrb1, Ccrl2, and Grk5 mRNA levels are significantly reduced in a bleomycin-induced mouse model that should be investigated further. The lack of changes in Cxcl13, Ccl11, Ccl19, Xcl1, and Cxcl5 mRNA levels may be due to differences in bleomycin-induced pulmonary fibrosis and IPF, although models of bleomycin-induced pulmonary fibrosis are the most common experimental models for investigating IPF.

A previous study reported that PADI4, IGFBP7, and GADD45A serve as biomarkers for IPF, which may contribute to the diagnosis of IPF (22). This is the first study to establish and validate a diagnostic model using eleven chemokine-associated genes (CXCL2, CCRL2, ARRB1, XCL1, GRK5, PPBP, CCL19, CCL13, CCL11, CXCL6, and CXCL13). Among the eleven CR-DEGs, some have been studied in lung injury. CXCL2 is significantly upregulated in mouse lung tissue in a bleomycin-induced model of pulmonary fibrosis, and 5-azacytidine (a DNA methyltransferase inhibitor) reduces CXCL2 expression (23). The neutralization of CXCL2 reduced hydroxyproline in the lung tissue of bleomycin-induced mice, but did not reduce lung neutrophil infiltration. Interestingly, stimulation with CXCL2 did not promote the proliferation of lung fibroblasts or collagen expression (20). In our study, CXCL2 expression levels were inversely correlated with lung function parameters. High expression of CXCL2 in alveolar lavage fluid cells from patients with IPF suggests a poor prognosis. Interestingly, CXCL2 expression is reduced in IPF lung tissue, possibly due to different extents across cell types.

CCRL2 is a seven-transmembrane protein expressed in epithelial cells, endothelial cells, and a variety of leukocytes. CCRL2 is upregulated under inflammatory conditions and recruits CXCR2-mediated neutrophils at sites of inflammation (24). Additional studies have demonstrated that CCRL2 deficiency worsens obesity and insulin resistance by increasing macrophage infiltration into adipose tissue (25). CCRL2 was downregulated in IPF samples, bleomycin-induced pulmonary fibrosis, and TGFβ1-induced fibroblast. ARRB1, initially thought to be a negative regulator of G-protein-coupled receptor signaling, has been shown to regulate cellular functions and is involved in various physiological processes, including inflammation, immune responses, and tumorigenesis (26, 27).

Deletion of Arrb1 significantly inhibited autophagy and induced neuronal apoptosis and necrosis in a model of cerebral ischemia (28). Autophagy is a highly conserved intracellular process involved in cellular degradation and recycling that has been found to delay the pathological progression of IPF (29). XCL1 and its receptor (XCR1) are significant regulators of dendritic and T-cell immune responses. For example, T cell-derived XCL1 contributes to intestinal XCR1^+^ DC activation and migration (30). Mice deficient in XCL1 or XCR1 have attenuated CD8^+^ T-cell responses and lack the ability to generate regulatory T cells. However, the role of XCL1-mediated immune responses in IPF has not been previously reported.

GRK5 has been reported to be highly expressed in the cardiac fibroblast. Nuclear translocation of GRK5 results in fibroblast activation. Fibroblast-specific deletion of GRK5 attenuates myocardial fibrosis and hypertrophy after chronic Ang II infusion or ischemic injury (31). GRK5 expression was significantly reduced in fibroblasts from the IPF cohort and TGFβ1-treated fibroblasts. Thus, the role of RGK5 in IPF pathogenesis warrants further investigation. PPBP, also known as CXCL7, is expressed by multiple cells, including platelets, neutrophils, natural killer cells, lymphocytes, and megakaryocytes (32). It has been found that CXCL7 regulates various processes, including glucose metabolism, mitogenesis, extracellular matrix and plasminogen activator synthesis (33). Proteomic analysis of serum extracellular vesicles showed that PPBP could represent a potential biomarker of liver fibrosis in patients with chronic hepatitis C (34). Recent evidence suggests that PPBP is upregulated in the serum of COVID-19 patients compared to influenza and serves as a potential biomarker for the severity of COVID-19 (35). The role of increased PPBP in IPF pathogenesis remains to be determined.

Our findings were consistent with those previously reported. CCL19 has been reported to be highly expressed in the lung tissue of patients with IPF, which facilitates the recruitment and accumulation of dendritic cells to fibrotic lung and sustains chronic inflammation, driving IPF development (36). Plasma CCL13 and CXCL13 are prognostic markers of IPF, and a higher concentration of CXCL13 is associated with higher all-cause mortality (37). CXCL6 has been reported to be upregulated in IPF-derived BAL. CXCL6 mRNA levels significantly increased on day 1 after bleomycin treatment, and then gradually decreased to normal levels. The inflammatory response induced by CXCL6 promoted the progression of pulmonary fibrosis. CXCL6 antibody neutralization attenuates early lung inflammation and prevents pulmonary fibrosis after bleomycin administration (38). Taken together, our findings support the ongoing assessment of the prognostic potential of chemokines as biomarkers in future IPF trials.

Increasing evidence suggests that immune cells are involved in the pathogenesis of IPF (39–42). Immune cells produce chemokines and cytokines that regulate fibroblast phenotypes. Alveolar macrophages and CD4^+^ T cells secrete CCL1, which promotes the differentiation of lung fibroblasts into myofibroblasts and contributes to pulmonary fibrosis (8). High levels of CD138 plasma cells have been demonstrated in the lung tissues of IPF patients and in mouse models of bleomycin-induced pulmonary fibrosis. Treatment of mice with bortezomib resulted in the depletion of plasma cells, which attenuated the development of bleomycin-induced pulmonary fibrosis, suggesting that plasma cells are essential effector cells in the pathogenesis of pulmonary fibrosis (43). In the current study, we also observed higher levels of plasma and macrophages (M0 and M2) in the IPF cohort than in the control cohort. Subtype analysis showed a decrease in dlco, fev1, and fvc in subtype 2, which indicated that patients with subtype 2 had more severe disease. Immune cell infiltration revealed increased levels of plasma cells and M0 macrophages in subtype 2 tumors. Further research is required to determine the exact mechanism by which plasma cells and macrophages promote IPF progression.

Despite this, several limitations of this study still exist. First, lung tissue specimens from patients with IPF were not available to validate the analysis, despite using a bleomycin-induced lung fibrosis model and a TGFβ1-induced cell model. Second, not only do the mRNA levels of hub genes need to be verified, but the expression levels of these genes also need to be confirmed to further understand the molecular mechanisms of IPF. Third, to gain a comprehensive understanding of the nature of chemokines and their receptors in IPF, especially their unique roles in different cell types, further functional and mechanistic investigations are necessary to determine the potential role of chemokine-associated genes in IPF pathogenesis.

In conclusion, our study established and validated a chemokine system-related eleven-gene signature in a diagnostic model for IPF. The model could serve as a diagnostic biomarker and contribute to a better understanding of the role of chemokines in IPF pathogenesis. Moreover, we validated hub gene expression levels by RT-qPCR in bleomycin-induced pulmonary fibrosis and a TGFβ1-induced cell model. Further studies are required to clarify these findings.

## Data availability statement

The original contributions presented in the study are included in the article/**Supplementary Material**. Further inquiries can be directed to the corresponding author.

## Ethics statement

The animal study was reviewed and approved by Ethics Committee of China Three Gorges University (Approval No. 2022B100A).

## Author contributions

TZ developed the concept and designed this study. TZ and XW carried out the data analysis. TZ, XW, and XZ contributed to animal and cell experiment. TZ, KY, XL and JN wrote and edited the manuscript. All authors contribute to the article and approved the submitted version.

## Supplementary material

Supplementary Figure 1 PPI network of CR-DEGs using the STING database.

Supplementary Figure 2 GO and KEGG enrichment analysis of CR-DEGs. **(A)** GO enrichment analysis of CR-DEGs, including BP. **(B)** KEGG enrichment analysis of CR-DEGs.

Supplementary Figure 3 Independent experiments of treatment of MRC-5 with TGFβ1 or TNFα for 24 h.
