# Supplementary material for: Identification and validation of chemokine system-related genes in idiopathic pulmonary fibrosis
Source: Front Immunol. 2023 Apr 14;14:1159856. doi: 10.3389/fimmu.2023.1159856 (PMC10140527; doi:10.3389/fimmu.2023.1159856)
Supplement: Supplementary Figure 1 — PPI network of CR-DEGs using the STING database. [file DataSheet_2.docx]

**Supplementary Material**

Identification and validation of chemokine system-related genes in idiopathic pulmonary fibrosis

Tianming Zhao^1†^, Xu Wu^1†^, Xuelei Zhao^2†^, Kecheng Yao^3^, Xiaojuan Li^1^, Jixiang Ni^1*^

**^*^ Correspondence:** Jixiang Ni, jxnee77@163.com

Supplementary Figures and Tables


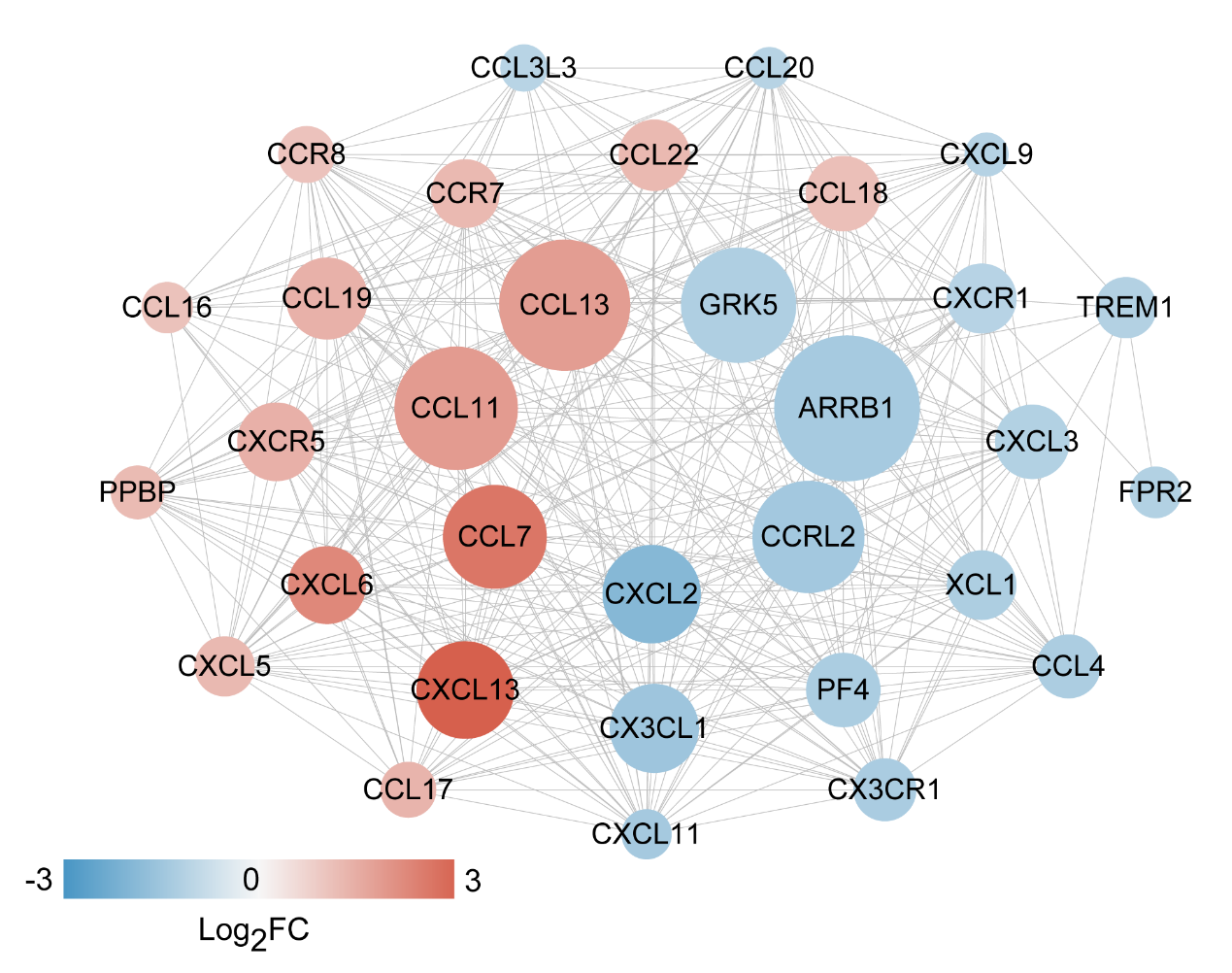


Figure S1. PPI network of CR-DEGs using the STING database


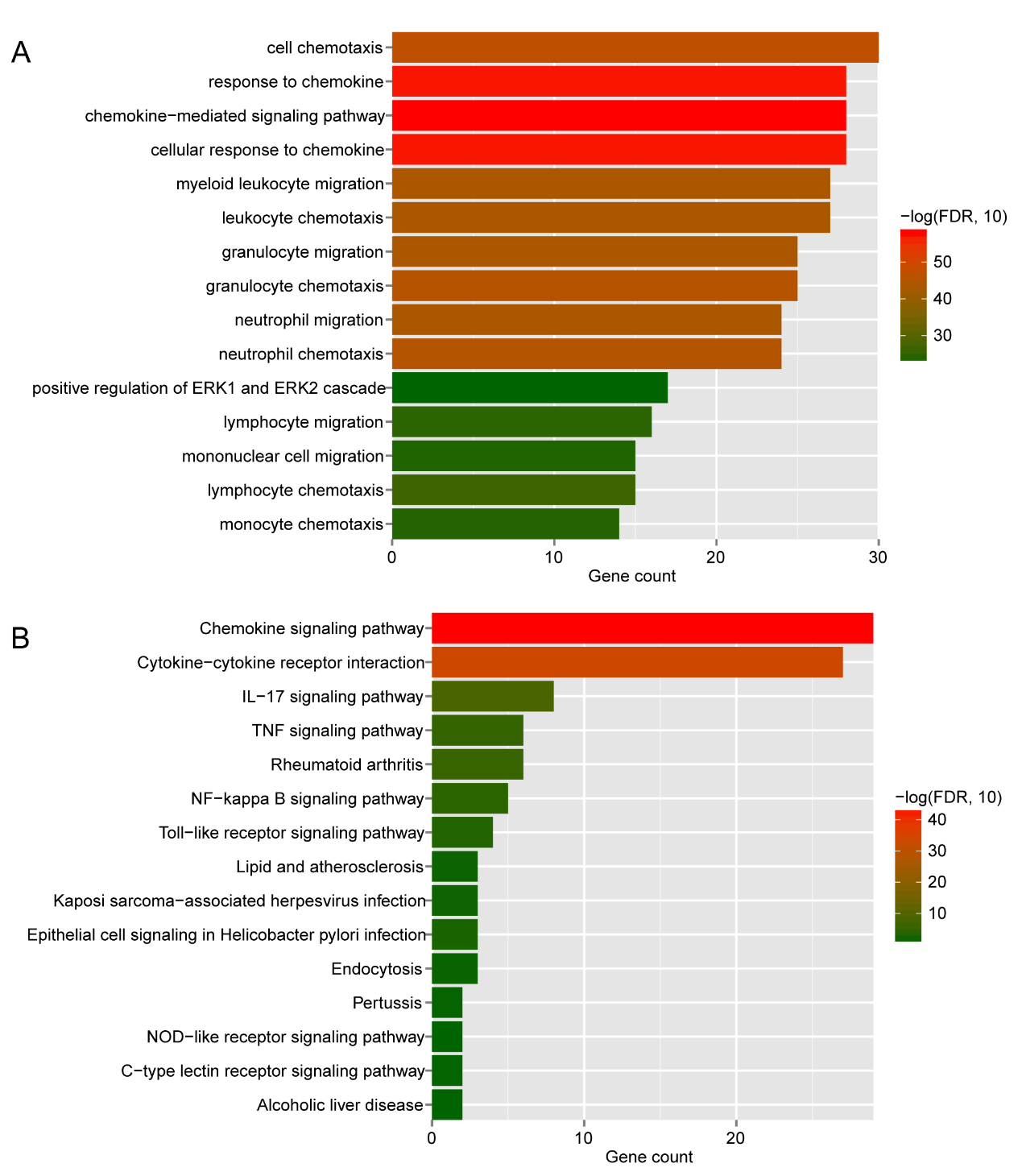


Figure S2. GO and KEGG enrichment analysis of CR-DEGs.

(A) GO enrichment analysis of CR-DEGs, including BP. (B) KEGG enrichment analysis of CR-DEGs.


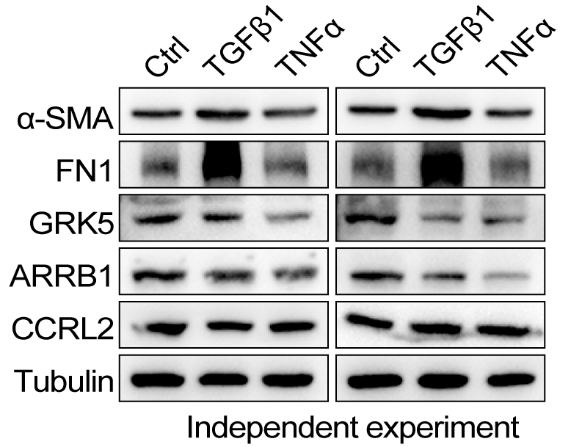


Figure S3. Independent experiments of treatment of MRC-5 with TGFβ1 or TNFα for 24 h.

Table S1 Chemokine related gene list.

| Gene Symbol | Description |
| --- | --- |
| ACKR1 | Atypical Chemokine Receptor 1 (Duffy Blood Group) |
| ACKR2 | Atypical Chemokine Receptor 2 |
| ACKR3 | Atypical Chemokine Receptor 3 |
| ACKR4 | Atypical Chemokine Receptor 4 |
| CCL5 | C-C Motif Chemokine Ligand 5 |
| CCL4 | C-C Motif Chemokine Ligand 4 |
| CCR7 | C-C Motif Chemokine Receptor 7 |
| CXCR3 | C-X-C Motif Chemokine Receptor 3 |
| CCR1 | C-C Motif Chemokine Receptor 1 |
| CX3CR1 | C-X3-C Motif Chemokine Receptor 1 |
| CXCL12 | C-X-C Motif Chemokine Ligand 12 |
| CCR5 | C-C Motif Chemokine Receptor 5 |
| CCR4 | C-C Motif Chemokine Receptor 4 |
| CCR6 | C-C Motif Chemokine Receptor 6 |
| CCR9 | C-C Motif Chemokine Receptor 9 |
| CCL15 | C-C Motif Chemokine Ligand 15 |
| CX3CL1 | C-X3-C Motif Chemokine Ligand 1 |
| CCL25 | C-C Motif Chemokine Ligand 25 |
| CXCR4 | C-X-C Motif Chemokine Receptor 4 |
| CCL19 | C-C Motif Chemokine Ligand 19 |
| CCR3 | C-C Motif Chemokine Receptor 3 |
| CCRL2 | C-C Motif Chemokine Receptor Like 2 |
| CCL21 | C-C Motif Chemokine Ligand 21 |
| PPBP | Pro-Platelet Basic Protein |
| CCR2 | C-C Motif Chemokine Receptor 2 |
| CCR8 | C-C Motif Chemokine Receptor 8 |
| CCL26 | C-C Motif Chemokine Ligand 26 |
| CCL20 | C-C Motif Chemokine Ligand 20 |
| CXCR6 | C-X-C Motif Chemokine Receptor 6 |
| CCL3 | C-C Motif Chemokine Ligand 3 |
| CCL23 | C-C Motif Chemokine Ligand 23 |
| XCR1 | X-C Motif Chemokine Receptor 1 |
| CCR10 | C-C Motif Chemokine Receptor 10 |
| CCL2 | C-C Motif Chemokine Ligand 2 |
| CCL18 | C-C Motif Chemokine Ligand 18 |
| CXCL13 | C-X-C Motif Chemokine Ligand 13 |
| CXCL10 | C-X-C Motif Chemokine Ligand 10 |
| CXCL2 | C-X-C Motif Chemokine Ligand 2 |
| CCL14 | C-C Motif Chemokine Ligand 14 |
| CXCL6 | C-X-C Motif Chemokine Ligand 6 |
| CCL16 | C-C Motif Chemokine Ligand 16 |
| CCL4L1 | C-C Motif Chemokine Ligand 4 Like 1 |
| CXCL11 | C-X-C Motif Chemokine Ligand 11 |
| CCL17 | C-C Motif Chemokine Ligand 17 |
| CXCL9 | C-X-C Motif Chemokine Ligand 9 |
| PF4 | Platelet Factor 4 |
| CXCL8 | C-X-C Motif Chemokine Ligand 8 |
| CCL3L3 | C-C Motif Chemokine Ligand 3 Like 3 |
| CXCL5 | C-X-C Motif Chemokine Ligand 5 |
| CCL22 | C-C Motif Chemokine Ligand 22 |
| CXCL3 | C-X-C Motif Chemokine Ligand 3 |
| CXCL1 | C-X-C Motif Chemokine Ligand 1 |
| CCL13 | C-C Motif Chemokine Ligand 13 |
| CCL8 | C-C Motif Chemokine Ligand 8 |
| CXCR2 | C-X-C Motif Chemokine Receptor 2 |
| CCL7 | C-C Motif Chemokine Ligand 7 |
| CXCR1 | C-X-C Motif Chemokine Receptor 1 |
| CCL11 | C-C Motif Chemokine Ligand 11 |
| CXCR5 | C-X-C Motif Chemokine Receptor 5 |
| XCL1 | X-C Motif Chemokine Ligand 1 |
| CCL1 | C-C Motif Chemokine Ligand 1 |
| CCL4L2 | C-C Motif Chemokine Ligand 4 Like 2 |
| CCL24 | C-C Motif Chemokine Ligand 24 |
| XCL2 | X-C Motif Chemokine Ligand 2 |
| CCL3L1 | C-C Motif Chemokine Ligand 3 Like 1 |
| CXCL16 | C-X-C Motif Chemokine Ligand 16 |
| HMGB1 | High Mobility Group Box 1 |
| PF4V1 | Platelet Factor 4 Variant 1 |
| IL4R | Interleukin 4 Receptor |
| CXCL17 | C-X-C Motif Chemokine Ligand 17 |
| ARRB1 | Arrestin Beta 1 |
| CCL27 | C-C Motif Chemokine Ligand 27 |
| ARRB2 | Arrestin Beta 2 |
| GRK6 | G Protein-Coupled Receptor Kinase 6 |
| FOXC1 | Forkhead Box C1 |
| PTK2B | Protein Tyrosine Kinase 2 Beta |
| C5 | Complement C5 |
| CMKLR1 | Chemerin Chemokine-Like Receptor 1 |
| GPR75 | G Protein-Coupled Receptor 75 |
| MCOLN2 | Mucolipin TRP Cation Channel 2 |
| CCL28 | C-C Motif Chemokine Ligand 28 |
| IL17A | Interleukin 17A |
| LYN | LYN Proto-Oncogene, Src Family Tyrosine Kinase |
| PAK1 | P21 (RAC1) Activated Kinase 1 |
| RAC1 | Rac Family Small GTPase 1 |
| CXCL14 | C-X-C Motif Chemokine Ligand 14 |
| TRPV4 | Transient Receptor Potential Cation Channel Subfamily V Member 4 |
| TSLP | Thymic Stromal Lymphopoietin |
| GRK5 | G Protein-Coupled Receptor Kinase 5 |
| CSF1R | Colony Stimulating Factor 1 Receptor |
| IL17F | Interleukin 17F |
| IL17RA | Interleukin 17 Receptor A |
| DOCK8 | Dedicator Of Cytokinesis 8 |
| TREM2 | Triggering Receptor Expressed On Myeloid Cells 2 |
| FFAR2 | Free Fatty Acid Receptor 2 |
| CARD9 | Caspase Recruitment Domain Family Member 9 |
| CHIA | Chitinase Acidic |
| PYCARD | PYD And CARD Domain Containing |
| TREM1 | Triggering Receptor Expressed On Myeloid Cells 1 |
| LGALS9 | Galectin 9 |
| FFAR3 | Free Fatty Acid Receptor 3 |
| ZC3H12A | Zinc Finger CCCH-Type Containing 12A |
| RIPOR2 | RHO Family Interacting Cell Polarization Regulator 2 |
| ITK | IL2 Inducible T Cell Kinase |
| PRKCA | Protein Kinase C Alpha |
| FPR2 | Formyl Peptide Receptor 2 |
| DOCK2 | Dedicator Of Cytokinesis 2 |
| GPR35 | G Protein-Coupled Receptor 35 |
| SH2B3 | SH2B Adaptor Protein 3 |
| GPR17 | G Protein-Coupled Receptor 17 |
| TFF2 | Trefoil Factor 2 |
| PLCG2 | Phospholipase C Gamma 2 |
| STAT1 | Signal Transducer And Activator Of Transcription 1 |

Table S2: Primer sequence

| Gene Symbol | Sequence |
| --- | --- |
| hACTB-F | ATCGTGCGTGACATTAAGGAGAAG |
| hACTB-R | AGGAAGGAAGGCTGGAAGAGTG |
| hARRB1-F | AAAGGGACCCGAGTGTTCAAG |
| hARRB1-R | CGTCACATAGACTCTCCGCT |
| hCCL11-F | CCCCTTCAGCGACTAGAGAG |
| hCCL11-R | TCTTGGGGTCGGCACAGAT |
| hCCL13-F | CTCAACGTCCCATCTACTTGC |
| hCCL13-R | TCTTCAGGGTGTGAGCTTTCC |
| hCCL19-F | CTGCTGGTTCTCTGGACTTCC |
| hCCL19-R | AGGGATGGGTTTCTGGGTCA |
| hCCRL2-F | AGCGATGAGGCAGAGCAATG |
| hCCRL2-R | GGACACCGATCACAAACACAG |
| hCXCL13-F | GCTTGAGGTGTAGATGTGTCC |
| hCXCL13-R | CCCACGGGGCAAGATTTGAA |
| hCXCL2-F | CCACACTCAAGAATGGGCAGAAAGC |
| hCXCL2-R | CCTCCTTCAGGAACAGCCACCAATA |
| hGRK5-F | CCAACACGGTCTTGCTGAAAG |
| hGRK5-R | TCTCTGTCTATGGTCCTTCGG |
| hPPBP-F | GTAACAGTGCGAGACCACTTC |
| hPPBP-R | CTTTGCCTTTCGCCAAGTTTC |
| hXCL1-F | TGCTCTCTCACTGCATACATTG |
| hXCL1-R | TGGTGTAGGTCTTGATTCTGCT |
| hCXCL6-F | AGAGCTGCGTTGCACTTGTT |
| hCXCL6-R | GCAGTTTACCAATCGTTTTGGGG |
| mGapdh-F | AGGTCGGTGTGAACGGATTTG |
| mGapdh-R | GGGGTCGTTGATGGCAACA |
| mCxcl2-F | CCAACCACCAGGCTACAGG |
| mCxcl2-R | GCGTCACACTCAAGCTCTG |
| mCcrl2-F | CCCCGGACGATGAATATGATG |
| mCcrl2-R | CACCAAGATAAACACCGCCAG |
| mArrb1-F | AAGGGACACGAGTGTTCAAGA |
| mArrb1-R | CCCGCTTTCCCAGGTAGAC |
| mXcl1-F | TAGCTGTGTGAACTTACAAACCC |
| mXcl1-R | ACAGTCTTGATCGCTGCTTTC |
| mGrk5-F | GAAAGCGCAAAGGGAAAAGCA |
| mGrk5-R | TCCTTCGGAGGTCTTCACACT |
| mPpbp-F | CTCAGACCTACATCGTCCTGC |
| mPpbp-R | GTGGCTATCACTTCCACATCAG |
| mCCL19-F | CCTGGGAACATCGTGAAAGC |
| mCCL19-R | TAGTGTGGTGAACACAACAGC |
| mCcl11-F | GAATCACCAACAACAGATGCAC |
| mCcl11-R | ATCCTGGACCCACTTCTTCTT |
| mCxcl5-F | GTTCCATCTCGCCATTCATGC |
| mCxcl5-R | GCGGCTATGACTGAGGAAGG |
| mCxcl13-F | GGCCACGGTATTCTGGAAGC |
| mCxcl13-R | ACCGACAACAGTTGAAATCACTC |

h: human, m: mouse, F: forward, R: reverse.
